# Supplementary material for: Bracing of Pectus Carinatum in Children: Current Practices
Source: Children (Basel). 2024 Apr 15;11(4):470. doi: 10.3390/children11040470 (PMC11049298; doi:10.3390/children11040470)
Supplement: Supplementary file 1 [file children-11-00470-s001.zip › children-2896583-supplementary.pdf]

Question	Answer categories
1. Country	
2. Type of institution	academic hospital • non-academic hospital
3. Type of department	pediatric surgery • thoracic surgery • pediatrics • other (specify)
4. Position	trainee • consultant • head of department
5. Number of new patients with pectus carinatum per year	≤ 25 • 26 – 50 • 51 – 75 • 76 – 100 • ≥ 101
6. Do you consider brace-treatment for pectus carinatum patients as the first-line option?	yes • no
7. What is your overall experience with pectus carinatum external bracing?	≤ 3 years • 4 – 6 years • 7 – 9 years • ≥ 10 years
8. How many patients have you treated with external bracing successfully so far?	≤ 50 • 51 – 100 • 101 – 150 • 151 – 200 • ≥ 201
9. Who is part of the therapeutic team? (more than one option can be selected)	surgeon • orthopedist • clinical anthropologist • physiotherapist • psychologist
10. What do you consider as the lowest age indication for external bracing?	≤ 8 years • 9 - 10 years • 11 - 12 years • 13 – 14 years • ≥ 15 years
11. What do you consider as the contraindication for brace treatment (more than one option can be selected)	cardiovascular diseases • connective tissue diseases • non-compressible chest deformity • other (specify)
12. Which patients are indicated for surgical intervention? (more than one option can be selected)	failure of conservative treatment • non-compressible chest deformity • rejection of brace treatment
13. What pre-treatment diagnostic modalities do you use routinely? (more than one option can be selected)	cardiological workup • spirometry • X-ray • CT scan • 3D optical scanner • clinical anthropometry • photography
14. What diagnostic methods do you use to determine the improvement of chest configuration during the treatment? (more than one option can be selected)	clinical examination • X-ray • CT scan • 3D optical scanner • clinical anthropometry • photography • other (specify)
15. Do you assess the pressure of correction/pressure of treatment?	yes • no
16. If yes, which pressure measurement device do you use?	(specify)
17. Recommended time for external brace wearing is	≤ 8 hours daily • 9 - 12 hours daily • 13 - 17 hours daily • ≥ 18 hours daily
18. Real, patient confirmed time for external brace wearing is	≤ 8 hours daily • 9 - 12 hours daily • 13 - 17 hours daily • ≥ 18 hours daily
19. Do you recommend external brace wearing even at night?	yes • no • it does not mater
20. How often do you perform out-patient controls during external bracing?	more frequent than once per 3 months • approximately every 3 months • less frequent than every 3 months
21. When do you consider to change the treatment to the maintenance (retainer modus) phase?	depends on the result of: anthropologic measurements • 3D optical scans • X-ray image • the patient´s satisfaction
22. When do you consider the treatment to be finished?	depends on the result of: anthropologic measurements • 3D optical scans • X-ray image • the patient´s satisfaction
23. How long does the brace treatment last on average?	≤ 9 months • 10 - 12 months • 13 - 15 months • 16 – 18 months • ≥ 19 months
24. Have you noticed a recurrence in puberty if brace treatment was indicated before the pubertal growth spurt?	no • yes in 1-10% • yes, in 11-20% • yes, in 21-30% • yes, in 31-40% • yes, in more than 41
25. Do you follow-up patients treated with external brace until the end of growth?	yes • no
26. What is the most common complication during brace treatment?	pain • rash • oedema • itching • other (specify)
27. What is the most common reason for treatment failure?	patient non-cooperation • complications • chest rigidity • other (specify)
28. What is the percentage of non-compliance patients?	≤ 10 % • 11 – 20 % • 21 – 30 % • 31 – 40 % • ≥ 41%
29. Do you use the treatment satisfaction questionnaire for your patients?	yes, during the treatment • yes, after the treatment • no
30. Is the costs for external brace covered by public health insurance?	fully paid • partly paid • not paid at all, the patient pays for the aid himself

File S1: Pectus Carinatum Brace Treatment Survey Questionnaire
